# Virtual field trips as physically active lessons for children: a pilot study

**DOI:** 10.1186/s12889-015-1706-5

**Published:** 2015-04-11

**Authors:** Emma Norris, Nicola Shelton, Sandra Dunsmuir, Oliver Duke-Williams, Emmanuel Stamatakis

**Affiliations:** Department of Epidemiology & Public Health, University College London, 1-19 Torrington Place, London, WC1E 7HB UK; Department of Clinical, Educational and Health Psychology, University College London, 26 Bedford Way, London, WC1H 0AP UK; Department of Information Studies, University College London, Foster Court, London, WC1E 6BT UK; Charles Perkins Centre, University of Sydney, Sydney, Australia; Exercise and Sport Sciences, Faculty of Health Sciences, University of Sydney, Sydney, Australia

**Keywords:** Virtual Field Trips, Physically active lessons, Physical activity, Schools

## Abstract

**Background:**

The modern classroom is an inherently sedentary environment. Virtual Field Trips (VFTs) using interactive whiteboards to explore virtual scenes are a potential method of converting sedentary class-time into physically active teaching. This pilot aimed to assess the effects of a developed VFT on physical activity and learning in primary-school children.

**Methods:**

Participants (n = 85) were randomly assigned to a) a 30-minute physically active London 2012 Olympics-themed VFT, or b) a 30-minute sedentary version of the same VFT. Activity was measured using GT1M Actigraphs, content recall was assessed with a quiz and user evaluations were gained from teacher and pupil questionnaires.

**Results:**

Pupils in the active VFT displayed significantly less sedentary time (p < 0.001), and significantly more light (p < 0.001), moderate (p = 0.01) and vigorous physical activity (p < 0.001) than sedentary VFT pupils. No differences in content recall were found between intervention groups: suggesting that adding physical activity into classroom teaching may not compromise attainment. High acceptability was found in teachers and active VFT students rated their session significantly higher than sedentary pupils (p < 0.002).

**Conclusions:**

This one-day pilot provides early evidence of the ability of VFTs to convert sedentary academic time into active time. Longitudinal research is needed to assess prolonged effects of active VFTs in reducing sedentary time.

## Background

Children currently spend 7–8 hours a day being sedentary [1], with most of this time spent in obligatory seated school lessons [2]. Children additionally do not perform more activity outside school hours to compensate for this inactivity [3], leading to an inherently inactive lifestyle and children unable to reach the recommended 60 minutes of moderate to vigorous exercise (MVPA) a day [4]. Current school inactivity is also undoubtedly a contributor to high rates in childhood obesity: with 33.3% of children currently overweight or obese [5]. Urgent action is needed to make school-time more active.

Schools are an important setting to promote physical activity (PA), allowing a large number of children to be exposed to interventions over a long period of time [6]. Although found to be largely effective in increasing active time [6,7], school PA interventions are often difficult to implement. They frequently involve securing time for PA outside of academic lessons, making them difficult for teachers to implement around academic priorities [8]. Integrating physical activity into educational time within classroom environments is one potential way of minimising such barriers for teachers and schools [9].

Physically active lessons are one way of doing this: promoting understanding of curriculum concepts via physical actions [10,11]. Such interventions emerge from the Social Ecological model, which recognises health behaviours such as physical activity as determined by both intrapersonal behavioural factors and interrelationships between individuals and wider social, physical and policy environments such as school peer-groups [12,13]. Projects such as Take 10! [14] and Physical Activity Across the Curriculum (PAAC) [15,16] integrate activity into short sessions of Maths, English and Social Sciences [17]. An example activity would be recalling multiplication tables whilst skipping or running [18]. Significantly improved PA levels have been found following physically active lessons [16,19], with corresponding improvements in educational outcomes [16,20,21]. These findings are supported by a wealth of other research finding physical activity to improve cognitive outcomes [22,23], on-task behaviour [24] and wellbeing [25] in children.

Classroom technology has untapped potential as a source of physical activity. With over 70% of classrooms now featuring interactive whiteboards [26], Virtual Field Trips (VFTs) using these multi-modal devices may be viable as a physically active lesson format. VFTs allow pupils to interact with virtual maps, landmarks and objects to gain multi-modal information and facilitate multiple learning styles [27]. Until now VFTs have been entirely sedentary and mostly restricted to development for university-level study [28,29]. However, given the inherently explorative and geographical nature of VFTs, they seem prime candidates as physically active lessons for school-aged children. Children could ‘cycle’, ‘run’ or ‘fly’ on-the-spot through virtual scenes embedded with educational elements whilst still being in their classroom. No research has yet assessed the potential of school-based VFTs to improve children’s physical activity levels.

This pilot study investigated the effects of a one-off Olympic-themed VFT on pupil’s physical activity. The study aimed to:

1. Objectively measure children’s physical activity during the VFT lesson and the school day
2. Assess VFT content recall
3. Assess user evaluations after physically active VFT sessions

## Methods

### Participants and study design

Pupils (n = 85) from four Year 5 classes (aged 9 to 10) from two London state-funded primary schools participated in the study. A 2×2 between subjects experimental design was used.

Cluster randomisation was performed at class level in each school, with one class randomly allocated to the ‘active’ intervention VFT condition, and the other to the ‘sedentary’ control condition.

### Instrumentation

**Figure 1:**
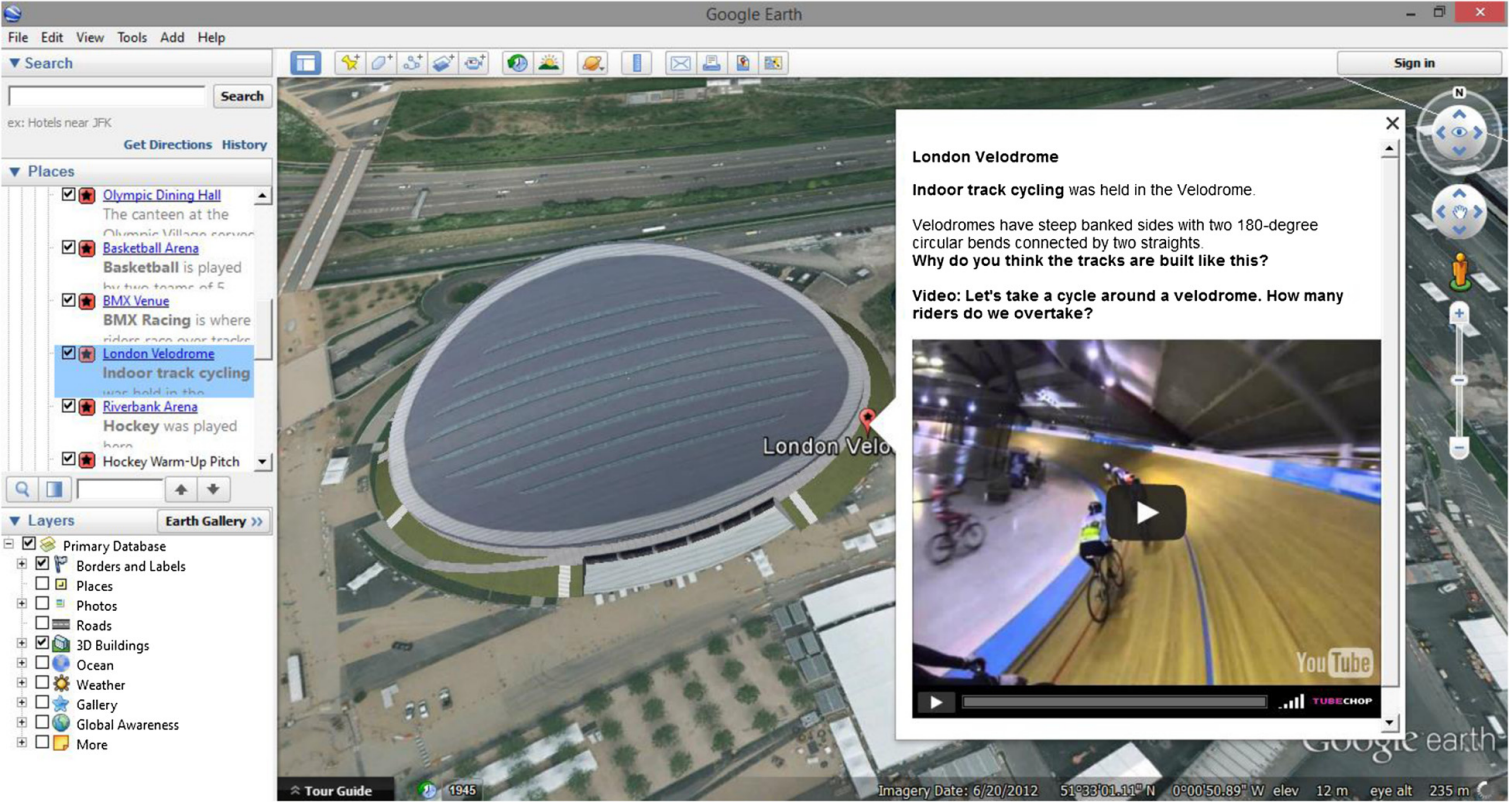
Example of developed Olympic-themed Virtual Field Trip with activity prompt.

A teacher-operated VFT was created using Google Earth: a free, widely available virtual globe [30], already available in the local software systems of participating schools. A London 2012 Olympics theme was chosen for the trip, due to the event’s recent and inherently active nature. Using the Interactive Whiteboard, the session involved navigating through various Olympic venues to discover more about their associated sporting events and was developed by the principal researcher (Figure 1). Existing 3D models of Olympic buildings made by other Google Earth users were used, with place marks, facts and multimedia content added using HTML. Questions related to sports hosted at each site were indicated with bold font. Both groups completed the session instead of a Topic (geography or history) lesson. Both VFTs featured the same building-specific information; however the intervention trip also included activity prompts in bold font to promote simulated exercises relating to each location. Intervention pupils stood throughout the 30-minute session, completing prompted activities such as running the 100 m finals on-the-spot in the Olympic Stadium or flapping their arms when ‘flying’ to the next location. Sedentary VFT participants were seated throughout the session and completed no related activities.

### Demographics

A questionnaire pack was sent to parents requesting their child’s gender, ethnicity [31] and whether they had watched the London Olympics at a venue or on television.

### Anthropometry

Weight was assessed by the lead researcher to the nearest 0.1 kg (Weight Watchers 8961U electronic scales, Milton Keynes, UK) and height to the nearest mm (2 metre tape measure). Body Mass Index (BMI; kg÷ m^2^) was then produced from these measurements. Underweight, overweight and obesity prevalence was estimated using the 2^nd^, 85^th^ and 95^th^ percentiles of the 1990 UK reference curves [32].

### Physical activity

Physical activity was measured from 9:00 AM to 3:00 AM during the study day using bi-axial Actigraph GT1M accelerometers (CSA, Shalimar, FL) worn on pupils’ waist above the right hip. Data was recorded using 15 second epochs, with this interval validated to measure bursts of activity typical in children [33].

### Learning

A ten-item quiz on content recalled from the Olympic VFT was issued to participants following the session. Four multiple choice questions were included, such as “When did the Olympic flame stay lit until? A: The Olympic Games closing ceremony, B: The end of 2012, C: The end of the men’s 100 m sprint final”. Six open-response questions were included, such as “What type of sport was held at the Velodrome?”

### Process evaluation

Evaluations sheets were supplied to participants and teachers after the VFT. This asked for their overall liking of the session (out of 5), whether they would like to take another VFT in the future (yes/no) and for positive and negative comments about the session. Difficulties in the provision of VFTs or outcome measurement were noted by the principal researcher.

### Procedure

The study was conducted during May and June 2013, with the pilot intervention run for one day in each class. Participants had anthropometric measurements taken on the morning of the study. The 30-minute VFT was delivered after the school lunch break, allowing one hour of post-VFT activity measurement time for this pilot. Post-VFT content recall and process evaluation forms were provided after the session. The session was delivered by the class teacher following a short briefing from the researcher. Pupils were blinded to their study condition but teachers were not to allow them to deliver their allocated session effectively. Ethical approval was granted from University College London. Consent was obtained from participating children and their parents, as well as teachers.

### Statistical analysis

Actigraph data was downloaded immediately after each study day using ActiLife software version 6.0 (Actigraph, LLC, Fort Walton Beach, Florida). Pulsford cut-points [34] were applied to assess activity intensity (Sedentary: <100 counts per minute (CPM), light: 100–2240 CPM, moderate: 2241–3840 CPM, vigorous: ≥3841 CPM). Pulsford cut-points are calibrated from free-living as opposed to treadmill activities in children: reflecting the sporadic movements initiated by children and targeted to be elicited during VFT participation [33,34]. Accelerometer data was then extracted to SPSS (version 19; Chicago, SPSS Inc.) for analysis.

Missing demographic data was evident in parent questionnaires (n = 6, 5.1%) and missing pupil VFT evaluations (n = 5, 4.25%). Pairwise deletion of missing activity was hence used to retain maximal data. Descriptive statistics of demographics, activity levels, post-VFT quiz learning outcome and VFT teachers and pupil evaluations were performed. Pearson chi-squared tests were used to assess demographic differences. One-way ANOVAs were used to assess differences in outcomes between intervention, school and demographic groups. Independent *t* tests were used to assess axes recordings across VFT groups. Multiple regressions were completed for post-VFT quiz learning outcome and sedentary, light, moderate and vigorous activities during the VFT period. Activity intensities of school day activity outside of VFT sessions were used in the VFT activity regression analysis. Dummy coding was used for categorical variables of ethnicity, BMI category and teacher. Backward stepwise entry was used, with all variables entered first before removing those non-significant. Models producing the greatest amount of explained variance from adjusted R^2^ were reported.

## Results

### Participants

Of the eighty five participants, 47.1% (n = 40) were allocated to the active, intervention VFT condition. 58.8% (n = 50) were male, 56.5% (n = 48) were white, 25.9% (n = 22) were Asian or Asian British and 5.9% (n = 5) were Black or Black British. 20% (n = 17) were obese and 8.2% (n = 7) were overweight. 8.6% (n = 7) had attended a London 2012 Olympics event at an official venue and 87.7% (n = 71) had watched the Olympics on television. Significantly more pupils watched the Olympics on television in the sedentary compared to the active condition groups (*χ*^2^(1) = 4.28, p < 0.05). There were no other significant differences in demographics between intervention groups and schools (Table 1).

**Table 1:** **Descriptive characteristics of 85 participants**

**Demographics**	**All (N = 85)**	**Active VFT (N = 40)**	**Sedentary VFT (N = 45)**	**p**
**Gender**				
Male	58.8% (n = 50)	57.5% (n = 23)	60% (n = 27)	0.82
Female	42.2% (n = 35)	42.5% (n = 17)	40% (n = 18)	
**Ethnicity**				
White	56.5% (n = 48)	55% (n = 22)	57.8% (n = 26)	0.92
Asian	25.9% (n = 22)	30% (n = 12)	22.2% (n = 10)	
Black	5.9% (n = 5)	5% (n = 2)	6.7% (n = 3)	
Arab	2.4% (n = 2)	2.5% (n = 1)	2.2% (n = 1)	
Mixed	4.7% (n = 4)	2.5% (n = 1)	6.7% (n = 3)	
Other	2.4% (n = 2)	2.5% (n = 1)	2.2% (n = 1)	
Missing	2.4% (n = 2)	2.5% (n = 1)	2.2% (n = 1)	
**BMI (kg÷ m** ^**2**^ **) Mean (SD)**	18.37 (3.42)	18.27 (3.77)	18.46 (3.13)	*t(83) = −0.25, n.s
**BMI Category**				
Normal	71.8% (n = 61)	70% (n = 28)	73.3% (n = 33)	0.85
Overweight	8.2% (n = 7)	7.5% (n = 3)	8.9% (n = 4)	
Obese	20% (n = 17)	22.5% (n = 9)	17.8% (n = 8)	
**Watched Olympics at official venue**	8.6% (n = 7)	2.5% (n = 1)	13.3% (n = 6)	0.052
**Watched Olympics on TV**	83.5% (n = 71)	80% (n = 32)	86.7% (n = 39)	0.04

Note: Pearson chi-squared tests used to assess VFT condition differences, *indicates independent samples t-tests.

### Physical activity during VFT sessions

97.6% (n = 83) of participants had available accelerometer data. There were significant differences in physical activity during the VFT between intervention groups (Table 2). Active intervention pupils had significantly fewer sedentary bouts (M = 0.11, SD = 0.31) during the VFT than sedentary group pupils (M = 0.56, SD = 0.62) (F(1,82) = 16.35, p < 0.001) and completed less sedentary time (M = 28.90 mins, SD = 5.12) than the sedentary group (M = 34.88 mins, SD = 7.80) (F(1,82) = 16.35, p < 0.001). The majority of time in both groups was recorded as sedentary by accelerometers (active 63.9%; sedentary 76.3%), despite active VFT participants standing and moving throughout the session. Active intervention pupils engaged in significantly more light activity (M = 14.97 mins, SD = 6.18) than the sedentary group (M = 9.92 mins, SD = 6.11) (F(1,82) = 13.92, p < 0.001), more moderate activity (M = 1.07 mins, SD = 0.81) than the sedentary group (M = 0.61 mins, SD = 0.80) (F(1,82) = 6.89, p = 0.01) and more vigorous activity (M = 0.79 mins, SD = 0.65) than the sedentary group (M = 0.27 mins, SD = 0.64) (F(1,82) = 13.62, p < 0.001). There were no significant differences in VFT activity between gender, BMI or ethnicity groups.

**Table 2:** **One-way ANOVAs of physical activity during VFT**

**Physical activity level**	**All (N = 83)**	**Active VFT (N = 38)**	**Sedentary VFT (N = 45)**	**p**
**Sedentary Bouts**	0.35 (0.55)	0.11 (0.31)	0.56 (0.62)	<0.001
**Sedentary Time (mins)**	32.14 (7.31)	28.90 (5.12)	34.88 (7.80)	<0.001
**Light Time (mins)**	12.23 (6.61)	14.97 (6.18)	9.92 (6.11)	<0.001
**Moderate Time (mins)**	0.82 (0.84)	1.07 (0.81)	0.61 (0.80)	0.01
**Vigorous Time (mins)**	0.51 (0.69)	0.79 (0.646)	0.27 (0.64)	<0.001

Note: Mean in minutes (SD).

Multiple regression analyses were performed for activity intensities during the VFT (Table 3). 58.4% of sedentary VFT time was explained (F(9,75) = 39.33, p < 0.001), with sedentary VFT condition (p < 0.001) and more sedentary time during the day (p < 0.001) significantly associated with increased VFT sedentary time. 63.2% of light VFT activity was explained (F(6,82) = 24.46,p < 0.001), with school (p < 0.001), active condition (p < 0.001) and less sedentary time during the day (p < 0.001) significantly associated with more light VFT time. 7.8% of moderate VFT time was explained (F(2,77) = 4.25, p < 0.05). No included factors in the model were significantly associated with moderate VFT time, although active VFT condition approached significance (p = 0.053). 22.4% of vigorous VFT activity was explained (F(6,77) = 4.70, p < 0.001), with school (p < 0.05), active VFT condition (p < 0.001), girls (p < 0.05) and sedentary time during the (p < 0.01) significantly associated with increased VFT vigorous activity.

**Table 3:** **Backward multiple regression models of activity intensities during VFT with greatest adjusted R** ^**2**^

**DV**	**Step/predictor**	**B**	**β**	**DV**	**Step/predictor**	**B**	**β**
Sedentary Time	Condition	6.30	0.43***	Light Time	School	8.20	0.62***
	Sex	−1.65	−0.11		Condition	−5.16	−0.39***
	Sedentary Time during day	0.16	0.65***		Sedentary Time during day	−0.17	−0.77***
					Moderate Time during day	−0.16	−0.23
					Vigorous activity during day	0.08	0.11
F	39.33***			F	29.19***		
Adjusted R^2^	0.584			Adjusted R^2^	0.632		
**DV**	**Step/Predictor**	**B**	**β**	**DV**	**Step/Predictor**	**B**	**β**
Moderate Time	Condition	−0.37	−0.22*	Vigorous Time	School	0.39	0.28*
	Watched Olympics at venue	0.62	0.20		Condition	−0.49	−0.35***
					Sex	0.36	0.26*
					Watched Olympics on TV	−0.22	−0.11
					Watched Olympics at venue	0.40	0.15
					Sedentary time during day	−0.01	−0.33**
F	4.25*			F	4.70***		
Adjusted R^2^	0.078			Adjusted R^2^	0.224		

Note: Day activity intensities include all recorded time outside of VFT sessions; *p < 0.05; **p ≤ 0.01, ***p ≤ 0.001.

### Post-VFT physical activity

Post-VFT activity between intervention groups was analysed to assess the potential effects of active class sessions on subsequent school activity. VFT sessions were held following lunch breaks allowing only an hour of post-VFT measurement in this pilot study. Two classes (one intervention and one control) were permitted extended afternoon play after the VFT by their teachers due to good weather. To allow realistic assessment of the provisional effects of VFT on subsequent typical teaching, these two classes were removed from post-VFT physical activity analysis.

Following the VFT, the remaining active class demonstrated more moderate time (M = 0.88 mins, SD = 0.81) han the sedentary class (M = 0.55 mins, SD = 0.48) (F(1,38) = 9.19, p < 0.01). Conversely, significantly more vigorous time was found post-VFT in the sedentary class (M = 0.71 mins, SD = 0.46) compared to the active class (M = 0.15 mins, SD = 0.33) (F(1,38) = 18.30, p < 0.001). However these rates were extremely small in both groups and drawn from a reduced sample due to aforementioned external factors (Table 4).

**Table 4:** **One-way ANOVAs of physical activity after VFT in classes without extended afternoon play**

**Physical activity level**	**All N = 39**	**Active VFT N = 18**	**Sedentary VFT N = 21**	**p**
**Sedentary Bouts**	0.54 (0.64)	0.50 (0.62)	0.57 (0.68)	0.73
**Sedentary Time (mins)**	43.28 (5.56)	41.69 (5.74)	44.63 (5.16)	0.10
**Light Time (mins)**	14.42 (5.31)	15.89 (5.42)	13.15 (4.99)	0.11
**Moderate Time (mins)**	1.14 (0.69)	0.88 (0.81)	0.55 (0.48)	0.004
**Vigorous Time (mins)**	0.46 (0.49)	0.15 (0.33)	0.71 (0.46)	<0.001

Note: Mean (SD).

### Physical activity during the school day

Eight (9.6%) of participants with accelerometer data achieved ≥60 minutes MVPA during the school day. However these were exclusively from the two classes with teacher-permitted extended play post-VFT, hence not reflecting typical teaching. Forty five (54.2%) performed over 30 minutes MVPA, with thirty five of these (77.8%) from classes with extended post-VFT play. There were no differences in physical activity across the school day between intervention groups (Table 5).

**Table 5:** **One-way ANOVAs of physical activity during school day including VFT session**

**Physical activity level**	**All N = 83**	**Active VFT N = 38**	**Sedentary VFT N = 45**	**p**
**Sedentary Bouts**	2.54 (1.89)	2.26 (1.59)	2.78 (2.10)	0.218
**Sedentary Time (mins)**	198.44 (34.33)	196.30 (27.69)	200.26 (39.31)	0.604
**Light Time (mins)**	111.60 (25.33)	113.90 (21.51)	109.65 (28.26)	0.450
**Moderate Time (mins)**	21.49 (9.56)	21.41 (8.07)	21.56 (10.75)	0.947
**Vigorous Time (mins)**	14.26 (8.54)	15.11 (9.46)	13.54 (7.73)	0.409

Note: Mean (SD).

### Accelerometer counts

Total accelerometer axes counts recorded during the VFT were analysed to assess the types of movement elicited. The bi-axial GT1M model used detects axis 1 (Y-axis): reflecting accelerating, ambulatory movement such as running and axis 2 (X/ anteroposterior axis) reflecting vertical movements such as jumping [35]. Significantly more Y-axis counts were recorded from the active VFT group (16804.21 counts, SD = 9684.87) compared to sedentary group (8826.29 counts, SD = 7578.59) (t(81) = 4.21, p < 0.001). There were also significantly more X/A-P axis counts in the active (22308.53 counts, SD = 8635.34) compared to the sedentary group (11754.42 counts, SD = 7742.71) (t(81) = 5.87, p < 0.001). More counts were recorded on the X/ A-P axis than the Y axis by the active group, suggesting VFT-prompted activity produced more on-the-spot rather than ambulatory movement.

### VFT content recall

A mean score of 7.55 out of 10 (SD = 1.90) was attained across all participants for the post-VFT quiz. There were no significant differences in post-VFT quiz marks between intervention groups. This suggests there was no detrimental effect of physically active versus sedentary VFTs on learning in this pilot sample. Scores were fairly high across participants (M = 7.55 out of 10; SD = 1.90), which may suggest a potential ceiling effect. There were also no significant post-VFT quiz mark differences between genders, ethnicities, BMI categories and Olympic venue attendance. This suggests that learning via VFT may be beneficial for a diverse range of pupils. Participants who had watched the Olympics on television scored significantly higher (M = 7.71, SD = 1.78) than those who had not (M = 6.20, SD = 2.25) (F(1,79) = 5.98, p < 0.02).

A multiple regression analysis was performed for the post-VFT learning outcome using backwards stepwise entry (Table 6). 12.2% of variance in learning outcomes results was explained. This is a low degree of explained variance, suggesting unmeasured factors are also important. Pupils who watched the Olympics on television (p < 0.05) attaining significantly higher scores. Obese pupils performed significantly worse than normal weight (p < 0.05).

**Table 6:** **Backward multiple regression models of post-VFT learning outcome with greatest adjusted R** ^**2**^

**Step/predictor**	**B**	**β**
Gender	−0.42	−0.11
Asian	−0.55	−0.13
Mixed Ethnicity	−1.06	−0.12
Obese	−1.21	−0.26*
Watched Olympics on TV	−1.46	−0.25*
F	3.20	
Adjusted R^2^	0.12	

Note: *p < 0.05.

### Participant evaluations

Pupil evaluation forms found that 84.7% (n = 72) wanted to do another VFT in the future. Significantly more pupils in the active condition (97.4%; n = 38) wanted to do another VFT than the sedentary condition (75.6%; n = 34) (F(1,83) = 8.83, p < 0.005). Pupils in the active condition also rated the VFT session significantly better (M = 4.5, SD = 0.98) than the sedentary condition (M = 3.86, SD = 1.24) (F(1,79) = 6.52, p < 0.02). Pupils across both conditions commented that improvements could be made to make future trips more interactive and realistic.

Teacher evaluation forms found that all teachers (n = 4) wished to run another VFT session in the future, with ratings from 3 and over out of 5. Evaluations were highest in an active class where the teacher proactively reorganised the classroom to facilitate activity. Teachers praised the use of Google Earth software as being free, readily available and easy to use.

## Discussion

This pilot study assessed the effects of a one-day VFT intervention in primary-school classes. To the authors’ knowledge, it is the first study to examine VFTs as physically active classroom sessions. The aims were to assess the impact of this pilot VFT on physical activity and content recall as a learning outcome, as well as assessing pupil and teacher evaluations.

### Physical activity during VFT

Accelerometer data found that the active VFT intervention group engaged in significantly less sedentary time and more light, moderate and vigorous activity during the VFT than the sedentary group. Multiple regression analysis also found VFT condition to be a positive significant predictor of all PA intensities during the VFT. However, although pupils in the active intervention group were standing and visibly active throughout the session, 63.9% of the active group’s VFT activity was still recorded as sedentary. Such standing, on-the-spot activity clearly does not comply with officially defined sedentary behaviour: energy expenditure ≤ 1.5 metabolic equivalents and a sitting or reclining posture [36]. Although a thorough review of published child cut-points was performed to identify the most suitable available cut-points; accelerometers still detected the majority of active VFT activity as sedentary. This review found no cut-points specifically calibrated for non-locomotor, on-the-spot movements. Consequently, Pulsford cut-points [34] were selected as they feature calibration of sedentary through to VPA intensities and via non-treadmill, self-paced activity. Recording of active VFT time as sedentary is likely a consequence of accelerometers’ insensitivity in measuring non-ambulatory movements, such as cycling and on-the-spot movement in comparison to accelerating, travelling movements [37].

Given health and safety considerations in classroom environments, it is difficult to expect high amounts of MVPA during VFT sessions. Although MVPA was low (only 4.2% of the active VFT session), sedentary time was reduced in active VFT participants and largely replaced with objectively recorded light activity. Emerging research is investigating the benefits of converting school sedentary time to light activity time, via examples such as standing desks [38]. Accordingly, future VFT research could instead aim to displace sedentary with light intensity activity as a minimum. Behaviour change techniques such as goal setting and rewards [39] or gamification elements [40] could be applied in future VFT research to potentially add sustained activity improvement.

No significant differences were found between genders and ethnicities. Larger-scale research is needed to see if VFTs are effective at increasing activity in girls and ethnic minorities, who frequently demonstrate lower PA [41,42].

### School day physical activity

Whole school day activity did not significantly differ according to VFT condition: suggesting the session did not affect overall day activity levels. This may have been due to the afternoon timing of the session, as pupils’ activity was only recorded for around an hour after the VFT. Sample size for assessing activity during a typical school due was reduced as two classes were permitted extended play post-VFT by their teachers due to good weather. No participants with typical post-VFT teaching achieved ≥60 minutes MVPA during the school day, much lower than found in recent research [43]. As activity was only recorded during school time, it is unknown if VFT condition was associated with any differences in after-school leisure activity. As in previous studies [1,43], girls were found to be more sedentary than boys across the school day. Although girls engaged in equal VFT activity to boys, a gender bias is still evident in their lower overall school PA. Further study is needed to assess if VFTs as a novel physically active lesson can improve PA in girls.

### Content recall

Post-VFT content recall was assessed as another secondary outcome in this pilot study. There were no significant differences in post-VFT quiz results between intervention groups: suggesting no detrimental effects from the active VFT. This suggests that active VFTs could be integrated into teaching curriculum to improve physical activity and without compromising academic attainment. As there was no non-VFT control group, it is unknown if scores would have been lower or higher in classes without a novel VFT experience. Other studies have similarly found in-class activity sessions to cause neither deficit nor improvement to academic performance [43,44]. However the majority of evidence from reviews indicates positive associations between physically active class sessions and learning [21].

A potential ceiling effect was noted with high scores across participants. Although questions were provided across a range of difficulties, the recent nature of the Olympic subject matter may not have sufficiently challenged students. No pre-VFT questionnaire was provided to assess existing Olympic knowledge, as the primary outcome of this study was on feasibility rather than learning outcomes. However, future iterations should include a pre-assessment to better assess the impact of VFT on the learning outcome.

### Participant evaluations

Experiences of the principal researcher and evaluations of teachers and pupils identified active VFTs to be feasible in this study. Pupils in the active VFT condition rated sessions significantly better than those in the sedentary condition. The novelty of the active session as opposed to the more typical, seated class format of the sedentary VFT may have increased pupil enjoyment [43,45]. A range of teacher evaluations were found, with ratings from middling to upmost success. The highest ratings were given by a physically active VFT teacher who rearranged the class layout to allow more movement. Previous physically active classroom sessions have been delivered at-desk [20,24]. However, early evidence found here suggests that this extra effort may provide a more successful active VFT session for both teacher and pupils. Improvements of VFT software and class layout changes will be considered in the development of future sessions.

### Strengths and limitations

Accelerometers provided usable data and were acceptable by 97.6% participants in this pilot. However Pulsford et al. [34] cut-points used recorded the majority of active VFT group time as sedentary, despite participants standing and engaging in on-the-spot activity throughout. There is currently an absence of calibrated cut-points for on-the-spot activity. Future VFT research should be aware that accelerometers may record much of standing time as sedentary. Researchers could consider using non-ambulatory movement measurement devices such as inclinometers [46] if budgets allow.

Accelerometry data was only available for one day in this pilot study, meaning a novelty factor may have been present. Activity of reluctant or conversely over-enthusiastic pupils in this single session may have been less accurate results than measurement after multiple sessions [24]. Future, longitudinal study will explore whether the increased activity seen here can be maintained over more regular active VFTs. In this study, sessions were provided immediately after lunch-breaks, providing only around an hour of post-VFT activity assessment. Additionally, two classes were permitted extended post-VFT play by their teachers. This further reduces the ability of this pilot to understand the effects of VFTs on subsequent activity in typical teaching. Further study should provide VFTs earlier in the school day and set clear expectations for typical teaching to be otherwise enforced by teachers. The provision of VFTs longitudinally would also allow clearer assessment of their impact on activity during regular teaching arrangements. To be effective as PA interventions they must reduce sedentary time and sustain activity over repeated sessions without leading to compensation effects in subsequent reduced activity.

## Conclusions

This pilot research found active VFTs to be feasible for primary-school classrooms. An Olympic-themed VFT elicited reduced sedentary time and increased light, moderate and vigorous physical activity compared to a sedentary version. However, there was scope for improvement of VFT technology. These findings provide preliminary evidence into the potential of VFTs in primary-school classes. Longitudinal research is needed to assess whether VFTs can reduce sedentary time and improve PA in a larger sample and over a prolonged period of sessions.

## Abbreviations

VFT: Virtual field trip
MVPA: Moderate to vigorous activity
PA: Physical activity
CPM: Counts per minute
